# Phytosynthesis of Nickel Oxide Nanoparticles and Their Antioxidant and Antibacterial Efficacy Studies

**DOI:** 10.7759/cureus.58064

**Published:** 2024-04-11

**Authors:** Lakshana Suresh, Ramanathan Snega, P Geetha Sravanthy, Muthupandian Saravanan

**Affiliations:** 1 Department of Pharmacology, Saveetha Dental College and Hospitals, Saveetha Institute of Medical and Technical Sciences, Chennai, IND

**Keywords:** multidrug-resistant wound isolates, antioxidant, antibacterial activity, nickel oxide, green synthesis

## Abstract

Introduction: Multidrug-resistant (MDR) bacteria are widely acknowledged as a significant and pressing public health concern. *Tribulus terrestris *has been used as a health tonic in traditional medicine since ancient Vedic times. It was also utilized to synthesize small, well-dispersed metal nanoparticles (NPs). The biosynthesized nickel oxide nanoparticles (NiO-NPs) have a broad spectrum of biomedical uses.

Objective: The objective of the research was to utilize a green synthesis method to synthesize NiO-NPs using *Tribulus terrestris*, subsequently characterize, and this study aimed to assess the antioxidant and antibacterial effectiveness of these NPs against wound isolates that are resistant to multiple drugs.

Materials and methods: The synthesis of NiO-NPs was achieved through the titration method, which is a green synthesis approach, and it was characterized by using techniques such as ultraviolet-visible spectroscopy (UV), Fourier transform infrared (FT-IR), scanning electron microscopy (SEM), X-ray diffraction (XRD) analysis, and energy dispersive X-ray (EDX). The antioxidant activity of the NPs was evaluated using the 2,2-diphenyl-1-picrylhydrazyl (DPPH) assay, and antibacterial activity was done using the agar well diffusion method. IBM SPSS Statistics for Windows, Version 21 (Released 2012; IBM Corp., Armonk, New York, United States) is used for statistical analysis.

Results: The biosynthesized NiO-NPs exhibited a color change from dark brown to dark green, indicating the successful reduction of the NPs. UV analysis peaks were observed at 310-350 nm, while FT-IR analysis showed the peaks at various wavelengths such as 629.31cm^-1 ^(halo compound; C-Br stretching), 957.80cm^-1^(aromatic phosphates; P-O-C stretch), 1004.65cm^-1^ (aliphatic phosphates; P-O-C stretch), 1094.93cm^-1 ^(organic siloxane or silicone; Si-O-Si), 1328.38cm^-1 ^(dialkyl/aryl sulfones), 1604.88cm^-1 ^(open-chain azo-N=N-), 2928.68cm^-1^ (methylene C-H asym/sym stretch), 3268.65cm^-1 ^(normal polymeric "OH" stretch). The crystallinity of the NPs was determined to be 24.7%, while the remaining 75.6% exhibited an amorphous structure. The SEM image revealed a spherically agglomerated structure of the nano-ranged size NiO-NPs. The EDX analysis indicated the presence of elemental compositions Ni (7.4%), O (39.4%), and C (53.3%) in the biosynthesized NiO-NPs. These NPs demonstrated significant antibacterial activity against *Pseudomonas aeruginosa* and *Klebsiella pneumoniae*, moderate antibacterial activity against methicillin-resistant *Staphylococcus aureus* (MRSA), and the lowest antibacterial activity against *Enterococcus faecalis*.

Conclusion: Our in vitro results demonstrate that the biosynthesized NiO-NPs exhibit significant antioxidant and antibacterial activity. These NPs can be used as a future antimicrobial medication, particularly against MDR clinical wound isolates of *K. pneumoniae, P. aeruginosa,* and MRSA.

## Introduction

Nanotechnology is a rapidly advancing technology in contemporary times, with its applications extending across diverse scientific and industrial fields. A wide range of metals have been synthesized, including metal oxide nanoparticles (NPs) and silver, gold, platinum, magnesium, iron oxide, cesium oxide, and zinc oxide [1]. Among these synthesized NPs, nickel oxide nanoparticles (NiO-NPs) have drawn interest from scientists in a variety of disciplines due to their compact size, broadband gap, and semiconductor properties [2]. NiO-NPs have been successfully applied in several applications, including photocatalysis [3], bioanalysis [4], and magnetic materials [5]. The green synthesis method uses three main sources: Fungi, Bacteria, and autotrophs (plants and algae). Plant-mediated green synthesis is utilized in our current work because it is easy to use, widely accessible, safe for the environment, and nontoxic. A range of plant and microbial sources, such as the stem of *Berberis balochistanica* [6], extract from *Raphanus sativus*, and leaf extract from Sageretia thea [7,4], have been effectively utilized in the environmentally friendly production of NiO-NPs [8]. This research utilized *Tribulus terrestris*, a herbaceous plant that bears tiny leaves with sharp spines, and long, woody fruits. The fruit and root of the plant have been used medicinally in Ayurvedic and traditional Chinese medicine because of their phytochemical compounds (saponins, flavonoids, and alkaloids) and pharmacological qualities such as antibacterial [9], antifungal, anti-inflammatory [10], anticancer, antidiabetic, anti-Alzheimer, antileukemia, and anti-obesity effects [11]. The phytochemical compounds present in the plant extract function as reducing and capping agents, hence playing a vital role in the stabilization of NPs [12]. Notably, the plant’s fruits and leaves have the highest concentrations of total flavonoid and phenolic content as well as a strong antioxidant activity [13].

The green synthesized NiO-NPs have been found to exhibit antibacterial and fungicidal activity against a range of bacterial and fungal strains [14]. Research has shown that NiO-NPs are highly effective antibacterial agents against both Gram-positive and Gram-negative pathogens. The use of plant-mediated metal NPs against multidrug-resistant (MDR) bacteria reduces the resistance properties through mechanisms like disrupting the membrane potential and integrity of bacterial cells, preventing the formation of biofilms and reactive oxygen species (ROS), strengthening host immune responses, and blocking RNA and protein synthesis by inducing intracellular processes [15]. Various research studies have explored the green synthesis of NiO-NPs, including the utilization of *Tribulus terresteris* for NP synthesis. In our present research, we are synthesizing NiO-NPs using *Tribulus terrestris* leaf extract, which was characterized by Fourier transform infrared (FT-IR), field emission scanning electron microscopy (FE-SEM), energy dispersive X-ray (EDX), and X-ray diffraction (XRD) analysis. This study highlights the biomedical importance of the NiO-NPs, and they exhibited antioxidant and antimicrobial efficacy against MDR wound isolates. Due to its potential biological activities, it can be used for the treatment of various medical ailments.

## Materials and methods

Sample preparation

The *Tribulus terrestris* sample was obtained from HiMedia in a powder format. The plant’s aqueous extract was made using an autoclave-assisted method after 20 g of powdered plant extract was combined with 100 ml of distilled water. Using the titration method, 50 ml of 0.1M NiO was added dropwise to 100 ml of *Tribulus terrestris *extract, and the color change was observed after 24 hours of incubation. The color transformation indicated the synthesis of NiO-NPs, and then the sample was centrifuged to remove impurities. The biosynthesized NiO-NPs are lyophilized, stored in a screw-cap bottle for further characterization, and applied in various biomedical applications.

Characterization Method

UV-visible spectroscopy (Labman Double Beam UV-vis spectrophotometer LMSPUV1900S, India) was utilized to perform primary characterization of the synthesized NiO-NPs at 1 nm resolution to ensure the reduction of Ni^+^ ions to NiO-NPs using *Tribulus terrestris *as a reducing agent. Then, the UV-vis analysis was conducted to investigate the synthesis and stability of NiO-NPs, with measurements taken at T0 and T24 within the wavelength range of 200 to 1000 nm. UV-vis absorption spectra were recorded at 24 hours after incubating the NiO solution with the plant extract and distilled water used as a blank reference. Additionally, FT-IR analysis was carried out using the Bruker Alpha II instrument from Germany to examine the bonding between functional groups and the metal, focusing on the range of 500 to 4000 cm^-1^. The aim was to identify potential biomolecules involved in the capping of NiO-NPs and the reduction of metal precursors. The composition, size, and morphology of NPs were observed using FE-SEM (JEOL-800S), while the elemental composition of the NPs was determined through EDX analysis using the Oxford X-Plor-30/C-Swift instrument. The XRD (Bruker model D8 advance) is used to examine the atomic arrangement within crystals and involves conducting XRD analysis of the NPs. The sample was subjected to centrifugation at 10,000 rpm for 15 minutes to separate the components. The collected precipitate was then dried at 50 ͦ C in an oven to obtain the nickel pellets and was analyzed by the XRD unit.

Antibacterial activity

The biological activity of synthesized NiO-NPs against bacterial strains has been studied by using the agar well diffusion method. The wells were made on the agar plate which is about 6 mm, and the wells were filled with the NiO-NPs at different concentrations (20, 40, 60, 80, and 100 µg/ml) comparable to standard antibiotics against the Gram-positive (G+ve) bacteria (*Enterococcus faecalis* and methicillin-resistant Staphylococcus aureus (MRSA)) and Gram-negative (G-ve) bacteria *(Klebsiella pneumoniae,* and *P. aeruginosa*), respectively. The bacterial strains were swabbed onto the prepared Mueller Hinton Agar (MHA) plate, and streptomycin (30 µg/ml) was used as a control. Then, the Petri plates were incubated at 37℃ for 24 hours. The zonal rate of inhibition was observed after the 24-hour incubation.

Antioxidant activity

The antioxidant activity was carried out by the 2,2-diphenyl-1-picrylhydrazyl (DPPH) method. A 1 ml NiO-NPs at various concentrations ranging from 10 to 50 µg/ml was mixed with 1 ml of DPPH solution (1 mM in methanol) and thoroughly vortexed L-ascorbic acid was added to the DPPH solution as a positive control. The mixture was then incubated for 30 minutes at room temperature and was protected from light. The absorbance was measured at 517 nm using a UV-vis spectrophotometer. DPPH was used as the blank, where all reagents were used except for the sample. The free radical scavenging activity was expressed as a percentage, and the standard deviation was calculated using IBM SPSS Statistics for Windows, Version 21 (Released 2012; IBM Corp., Armonk, New York, United States) based on the mean of triplicate results.

## Results

Synthesis of NiO-NPs from *Tribulus terrestris*

**Figure 1:**
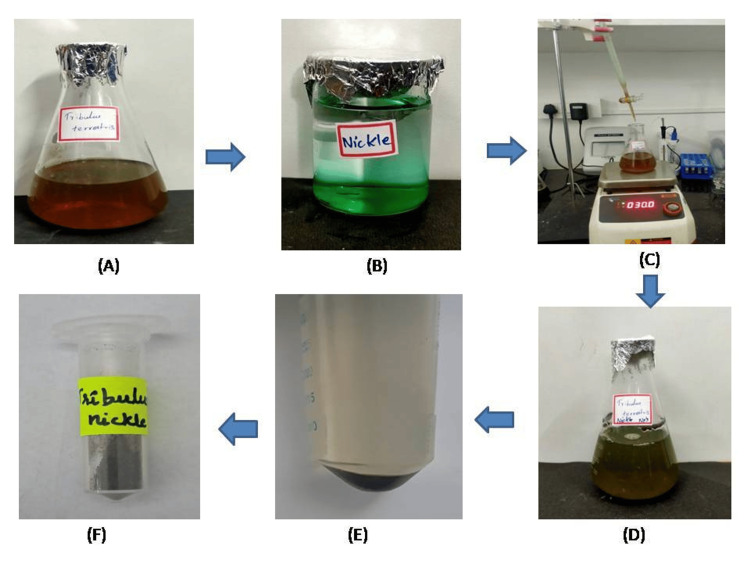
Tribulus terrestris-mediated synthesis of NiO-NPs by the titration method (green synthesis) NiO-NPs: Nickel oxide nanoparticles (A) *Tribulus terrestris* extract before synthesis, (B) nickel oxide solution, (C) titration method, (D) after 24-hour incubation NiO-NP synthesis, (E) centrifuge and pellet collection, and (F) NiO-NPs powder form

In this study, the green synthesis titration method was utilized to synthesize NiO-NPs by incorporating the aqueous extract of *Tribulus terrestris*. The successful synthesis of NiO-NPs was visually confirmed by the observed color transformation from dark brown to dark green, as illustrated in Figure 1.

UV-vis spectroscopy

**Figure 2:**
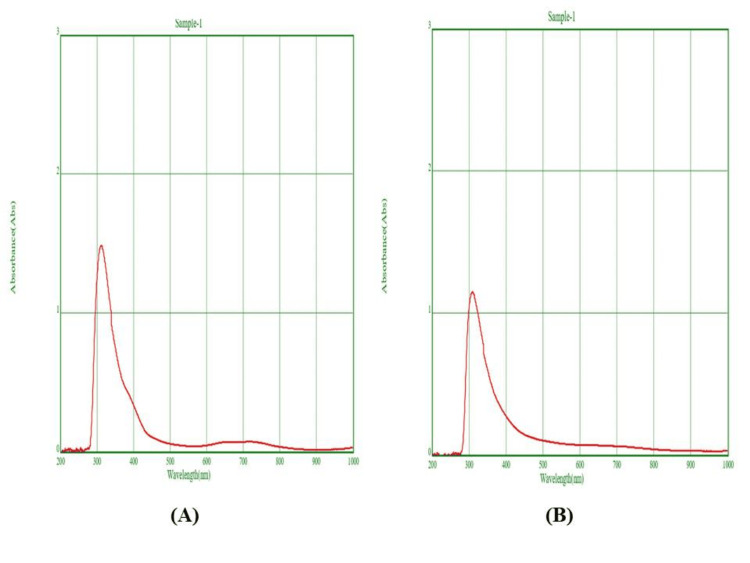
UV-vis absorption spectrum of the NiO-NPs UV-vis: UV-visible; NiO-NPs: nickel oxide nanoparticles (A) UV spectroscopy analysis-time 0 interval, (B) UV spectroscopy analysis-time 24-hour interval

The UV-vis spectroscopy analysis was conducted for the initial (T0) hour and after 24 hours (T24). The results showed that the peaks observed in the range of 310-350 nm confirmed the presence of biosynthesized NiO-NPs (Figure 2).

FT-IR

**Figure 3:**
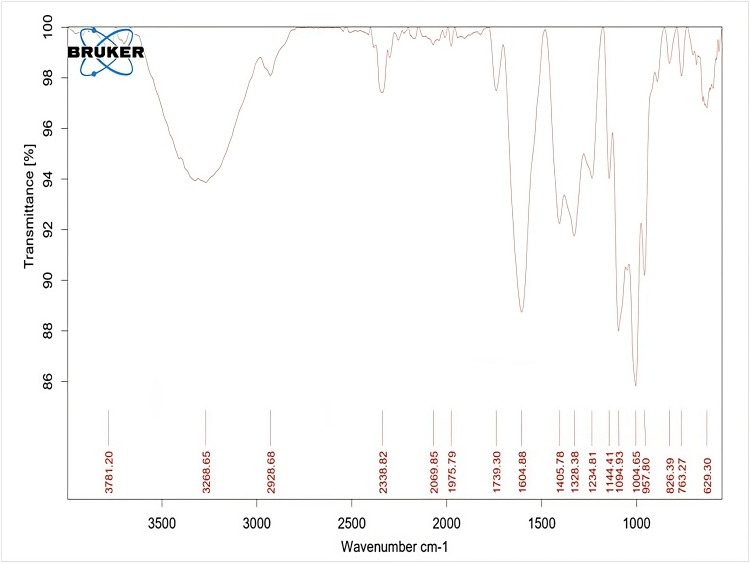
Fourier transformed infrared spectra of the synthesized NiO-NPs NiO-NPs: Nickel oxide nanoparticles

The biosynthesized NiO-NPs were characterized by FT-IR spectroscopy to identify the functional groups present in the synthesized NPs, and it showed more than four different functional groups present in the synthesized NPs. Figure 3 revealed the peak values and the occurrence of compounds such as 629.31cm^-1^ (halo compound; C-Br stretching) 957.80cm^-1^ (aromatic phosphates; P-O-C stretch), 1004.65cm^-1^ (aliphatic phosphates; P-O-C stretch), 1094.93cm^1^ (organic siloxane or silicone; Si-O-Si), 1328.38cm^-1 ^(dialkyl/aryl sulfones), 1604.88cm^-1 ^(open-chain azo-N=N-), 2928.68cm^-1 ^(methylene C-H asym/sym stretch), and 3268.65cm^-1 ^(normal polymeric "OH" stretch).

XRD

**Figure 4:**
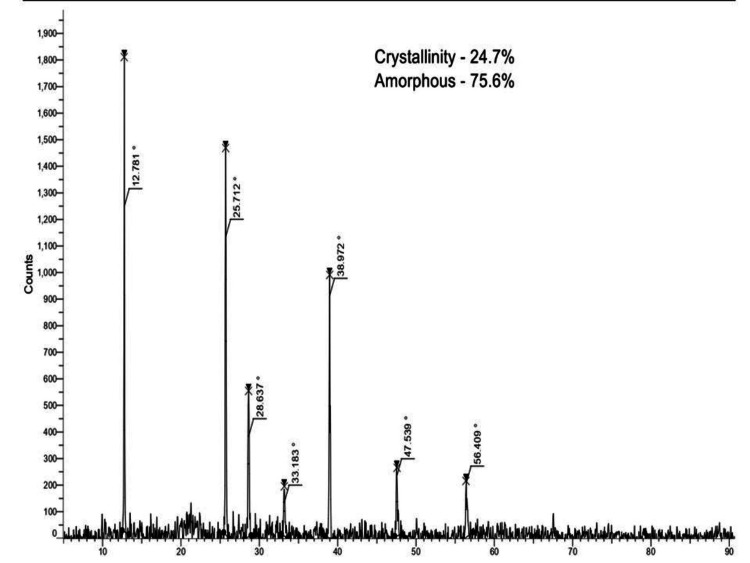
X-ray powder diffraction spectra of NiO-NPs NiO-NPs: Nickel oxide nanoparticles

XRD analysis is used to provide information about the crystalline structure and the size of particles. The XRD patterns of the synthesized NiO and NiO-NPs are displayed in Figure 4. The result has shown the diffraction peaks observed at the theta angles 12.781°, 25.712°, 38.972°, 47.539°, and 58.409°, and it revealed the crystallinity of 24.7% and the amorphous 75.6% structure.

**SEM** 

**Figure 5:**
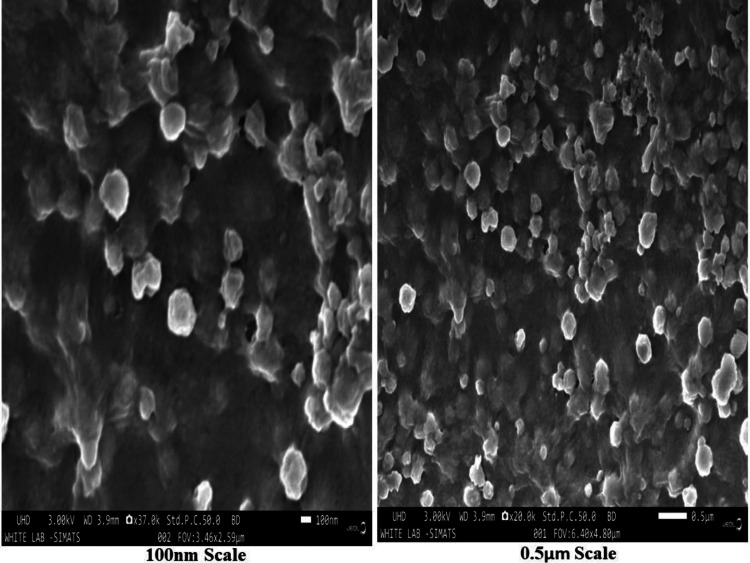
Scanning electron microscope images of the prepared NiO-NPs with different diameter distributions NiO-NPs: Nickel oxide nanoparticles (A) 0.5 μm scale, (B) 100 nm scale

The surface morphological characteristics of the fabricated nanoparticles were examined by SEM. The different (100nm and 0.5 μm) magnification ranges of the biosynthesized NiO-NPs results are shown in Figure 5. The SEM image revealed the nano-ranged size of the spherically agglomerated structure of NiO-NPs. 

EDX 

**Figure 6:**
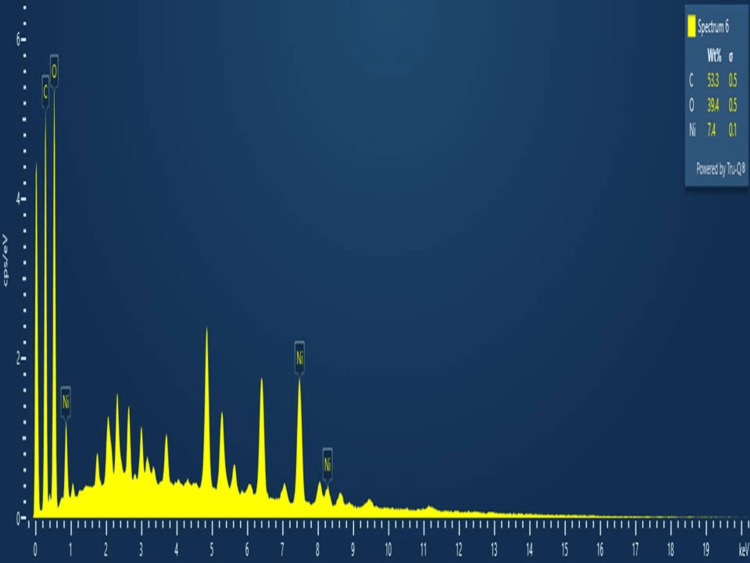
EDX images of biosynthesized NiO-NPs NiO-NPs: Nickel oxide nanoparticles; EDX: energy dispersive X-ray

EDX analysis was employed to determine the elemental composition and compounds present. According to Figure 6, the biosynthesized NiO-NPs exhibited peaks corresponding to Ni (7.4%), O (39.4%), and C (53.3%). The EDX peaks observed at 0.8 keV, 7.4 keV, and 8.2 keV were attributed to the presence of "Ni," while the other peaks at 0.4 keV indicated the presence of carbon (C), and the peaks at 0.6 keV indicated the presence of oxygen (O). 

Antibacterial activity 

**Figure 7:**
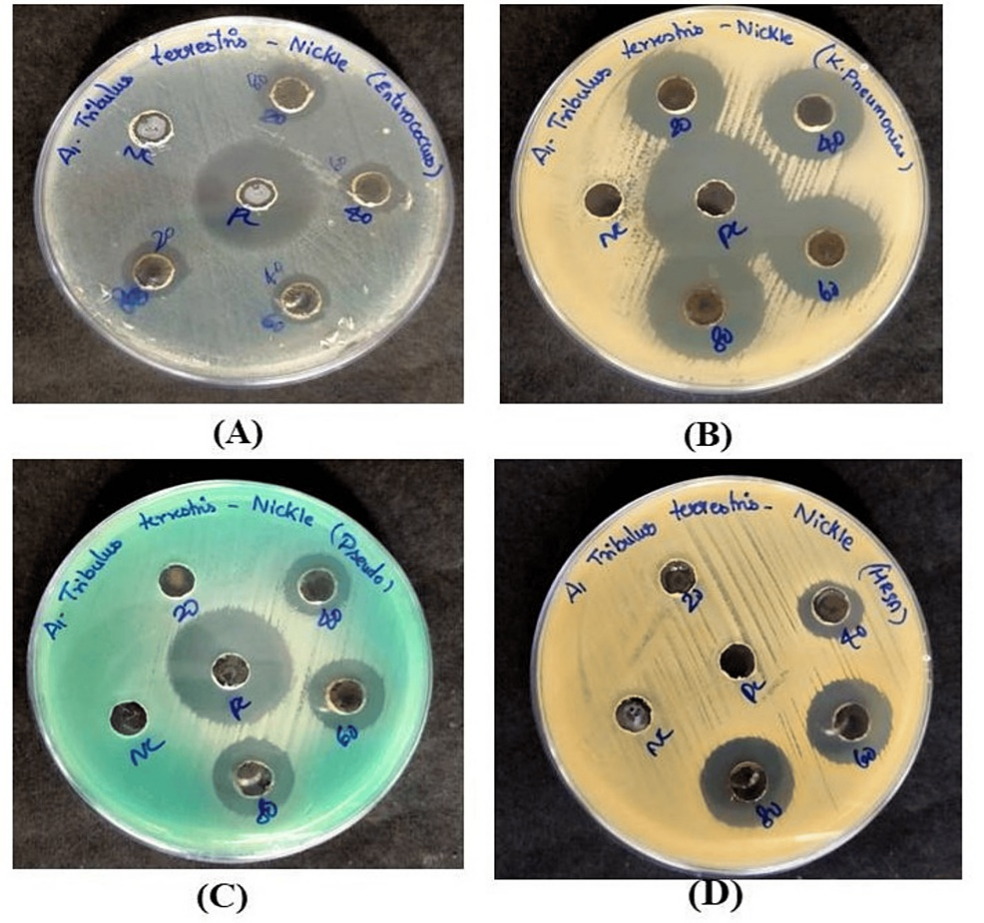
Antibacterial activity of NiO-NPs against the test organisms NiO-NPs: Nickel oxide nanoparticles (A) *Enterococcus faecalis*, (B) *Klebsiella pneumoniae*, (C) *Pseudomonas aeruginosa*, (D) methicillin-resistant *Staphylococcus aureus* (MRSA)

The antibacterial activity of biosynthesized NiO-NPs was assessed against *Enterococcus faecalis*, *Klebsiella pneumoniae*, *Pseudomonas aeruginosa,* and MRSA in a dose-dependent manner (20, 40, 60, and 80 μg/ml, respectively). Analysis of Figure 7 revealed the zone of inhibition demonstrated significantly higher antibacterial activity against *K. pneumoniae* (19 mm, 20 mm, 22 mm), *P. aeruginosa* (14 mm, 15 mm, 16 mm, 24 mm), and* E. faecalis *(13 mm, 12 mm, 12 mm) while displaying moderate antibacterial activity against MRSA (12 mm, 16 mm, 18 mm), respectively.

Antioxidant activity

**Figure 8:**
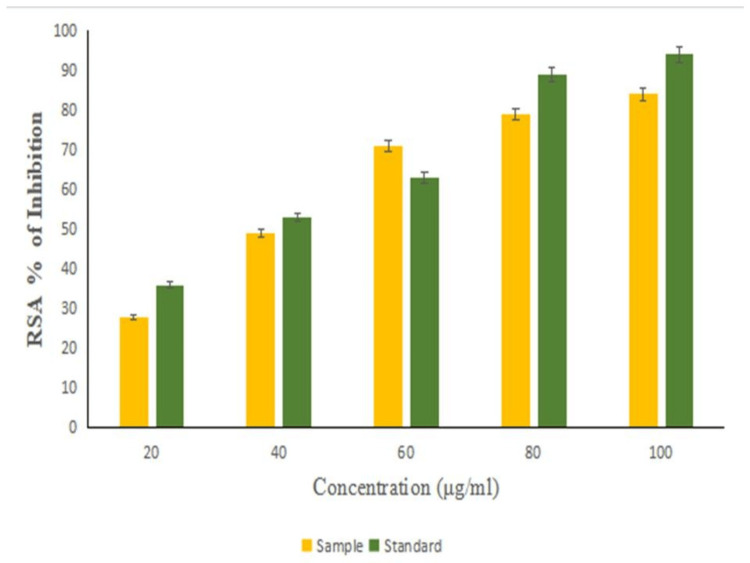
Antioxidant activity (DPPH) of NiO-NPs compared with the standard L-ascorbic acid DPPH: 2,2-diphenyl-1-picrylhydrazyl; NiO-NPs: nickel oxide nanoparticles

Figure 8 demonstrates the antioxidant activity of the biosynthesized NiO-NPs compared to the standard L-ascorbic acid in a dose-dependent manner. As the concentration of NiO-NPs increased (20, 40, 60, 80, and 100 μg/ml), the antioxidant activity also increased (28%, 49%, 69%, 71%, and 84%, respectively). The highest antioxidant activity (84%) was observed at the maximum concentration (100 μg/ml), while the lowest antioxidant activity (28%) was observed at the minimum concentration (20 μg/ml). Notably, at the optimum concentration of 60 μg/ml, the biosynthesized NiO-NPs exhibited a higher antioxidant activity (71%) compared to the standard drug (62%).

 

## Discussion

*Tribulus terrestris* is known for its abundance of bioactive components, such as saponins, flavonoids, and alkaloids. The synthesis of NiO-NP can be visually observed through a color change from dark green to dark brown, which is attributed to the reduction of NPs. The phytochemical compounds present in the plant serve as both reducing and capping agents in this process [16], and the reducing agent converts metal ions to their corresponding NPs and simultaneously acts as an effective capping agent to prevent the NP agglomerations as revealed by the literature [17].

In our investigation, the UV-vis spectroscopy demonstrated consistent peak ranges between 310 and 350 nm at both the T0 and T24. This indicates the rapid reduction and stability of the synthesized NiO-NPs. Similar peak ranges have been observed in previous studies. For example, prior research [18] reported an optimal absorbance of NiO-NPs at a wavelength of 350 nm. Another study [19], which utilized *Ageratum conyzoides* L leaf extract, showed a peak at 324 nm for the biosynthesized NiO-NPs. Based on these observations, our study showed similar peak ranges of NiO-NPs using *Tribulus terrestris*, and it can be attributed to the optical properties of the surface plasmon resonance.

The FT-IR analysis of the sample revealed the presence of various compounds. A peak observed at 3268.65 cm^-1^ indicated the presence of the OH group, consistent with findings from another study [20]. Additionally, a peak observed at 3480 cm^-1^ corresponded to the stretching vibrations of the O-H bond of H_2_O. The appearance of multiple absorption peaks could be attributed to the O-H bond of H_2_O. Furthermore, the peak at 629.31 cm^-1 ^was found to be linked with the formation of Ni-O bonds, as reported in previous studies [21,22]. Overall, the FT-IR data provided valuable information about the functional groups present, elucidating the involvement of biomolecules in the capping and dispersion of NiO-NPs.

The XRD analysis results indicate that the synthesized NiO-NPs have a crystallinity of 24.7% and an amorphous content of 75.6%. By comparing the XRD pattern with a standard sample, it was determined that the synthesized NiO-NPs possess a cubic crystalline structure (FCC) with a space group of Fm3m [23]. The presence of sharper peaks in the XRD patterns of high intensity and NiO-NPs suggests that the green synthesis method was successful in producing high-quality NiO. Furthermore, no additional peaks were observed in the XRD patterns, indicating the removal of all precursors and impurities, resulting in a pure NiO-NP sample. These findings are consistent with a previous investigation that reported the crystallographic structure of the NiO-NPs. Overall, the XRD analysis confirms the purity of the green synthesized NiO-NPs. The FE-SEM analysis revealed the presence of an agglomerated structure in various measurements. In comparison to a similar study, the biosynthesized NiO-NPs derived from the *Tribulus terrestris* fruit exhibited a spherically rod-shaped morphology with an agglomerated structure. This observation suggests that the fine rodlike particles tend to aggregate on the surface. Another study involving NiO-NPs synthesized using *Coriandrum sativum *leaf extract reported the presence of NP aggregates consisting of both nano- and microstructures, primarily attributed to magnetic interactions among the materials [24]. In our study, the EDX analysis indicated the presence of Ni (7.4%), O (39.4%), and C (53.3%). Comparing this with a similar study on biosynthesized NiO-NPs mediated by *Buxus wallichiana extract* [25], their EDX analysis showed Ni (75.6%) and O (20.9%). These findings confirm that the synthesized NiO-NPs in our study primarily consist of oxygen and nickel elements.

The antibacterial activity of the biosynthesized NiO-NPs exhibited significantly higher effectiveness against* E. faecalis* and* K. pneumoniae* while demonstrating moderate antibacterial activity against *P. aeruginosa* and MRSA. Comparing these results with a similar study, the bioactivity assay revealed that NiO-NPs displayed antibacterial activity against extended-spectrum beta-lactamase (ESβL), (+)* E. coli, P. aeruginosa*, and MRSA. The growth inhibition was observed through a time and concentration-dependent decrease in the viability of treated cells [19]. The mechanism of the antibacterial action of metal oxide NPs can be explained in various ways. In aqueous suspensions of NiO-NPs, the released Ni^+^ ions can interfere with the thiol group of essential bacterial enzymes, leading to inactivation and cell death. Additionally, the radicals present in the aqueous of NiO-NPs can interact with the negatively charged bacterial cell surface, disrupting vital life processes such as respiration and cell replication, ultimately causing inactivation and cell death [19].

In our current study, it was observed that NiO-NPs exhibited antioxidant activity of 84% at a concentration of 100 µg/ml. In comparison, the standard L-ascorbic acid demonstrated antioxidant activity of 94% at the same dosage. It was found that as the concentration of NiO-NPs increased, their ability to scavenge free radicals also increased. These findings align with a similar study [20], where NiO-NPs synthesized from the *Z. spina-christi* plant extract also demonstrated a significant antioxidant activity compared to the standard ascorbic acid using the modified DPPH method. Therefore, the results of our study confirm that the synthesized NiO-NPs exhibit novel antioxidant activity in our biological system.

Limitations

Our current study involved conducting a range of in vitro analyses to evaluate the NiO-NPs synthesized from plant extract. To gain a deeper understanding of its effects, additional in vivo research, such as animal and clinical trials, will be conducted.

## Conclusions

In conclusion, the study successfully demonstrates the environmentally friendly synthesis of biosynthesized NiO-NPs using the titration method, with the utilization of the medicinal plant *Tribulus terrestris* for green synthesis proving to be a highly advantageous approach. The characterization analyses, including XRD and FE-SEM, confirm the high crystallinity and unique structural properties of the NiO-NPs. Additionally, the EDX and XRD analyses validate the elemental composition and purity of the biosynthesized NiO-NPs. The significant antibacterial efficacy against various pathogens and strong antioxidant activity exhibited by the NiO-NPs highlight their potential for biomedical applications. Further research through in vitro and in vivo studies is essential to fully explore and harness the therapeutic potential of these biosynthesized NiO-NPs.
